# The who and what of validation: an experimental examination of validation and invalidation of specific emotions and the moderating effect of emotion dysregulation

**DOI:** 10.1186/s40479-022-00185-x

**Published:** 2022-05-18

**Authors:** Janice R. Kuo, Skye Fitzpatrick, Jennifer Ip, Amanda Uliaszek

**Affiliations:** 1 Department of Psychology, Palo Alto University, 1791 Arastradero Rd., Palo Alto, CA 94304 USA; 2 Department of Psychology, York University, Toronto, ON Canada; 3 Department of Psychology, Ryerson University, Toronto, ON Canada; 4 Department of Psychological Clinical Science, Scarborough, Toronto, ON Canada

**Keywords:** Validation, Invalidation, Emotion, Emotional reactivity, Emotion dysregulation

## Abstract

**Background:**

Theory and research indicate that validation is associated with reductions in negative emotions, whereas invalidation is associated with escalation of negative emotions. However, it remains unclear whether these effects are consistent across emotions, and/or moderated by an individual’s levels of emotion dysregulation. The present study experimentally examines the effects of validation and invalidation across emotions and as moderated by emotion dysregulation.

**Methods:**

One hundred twenty-six participants completed a measure of emotion dysregulation, and then listened to a rejection-themed imagery script after which they reported the intensity of several emotions. Participants were then presented with either validating or invalidating feedback about their most intense self-reported emotion, depending on their counterbalancing order. They then repeated the procedure for the other condition. Self-reported negative emotions via continuous rating dial, heart rate (HR), and skin conductance level (SCL) were monitored throughout.

**Results:**

Higher emotion dysregulation was associated with greater increases in self-reported positive emotion when shame or sadness was validated and lesser increases when fear was validated. There were no significant moderating effects of emotion dysregulation in response to invalidation for any emotion on any index.

**Conclusions:**

The effects of validation appear emotion specific and dependent on levels of emotion dysregulation. These findings may help inform more strategic use of validation in psychotherapeutic interventions.

**Supplementary Information:**

The online version contains supplementary material available at 10.1186/s40479-022-00185-x.

## Background

### Validation and psychotherapy

Validation, conceptualized by psychotherapists as “finding the truth in what we feel and think” [1], and the essence of which communicates to another that their responses make sense and are understandable within their life context or situation [2] is commonly applied in psychotherapy. Validation theoretically regulates client distress [3], fosters learning, and strengthens self-identity and therapeutic alliance [4]. Of note, Dialectical Behaviour Therapy (DBT, [2]) the gold-standard treatment for borderline personality disorder (BPD) and suicidal behaviour [5], operates on an overarching dialectic of balancing validation with behavioural change [2]. Within this model, DBT posits that the use of validation is core or fundamental to eliciting change.

### (In)validation and the impact on emotional reactivity

Although validation is posited to be a crucial technique in psychotherapy, there has been little empirical investigation into *how* or *why* validation works. One plausible mechanism is that validation decreases emotional reactivity, or the intensity or magnitude of emotional responses elicited by change in the internal or external environment [2, 3, 6]. Theory and emerging research suggest that validation functions to reduce negative emotional intensity, which subsequently fosters the learning and use of emotion regulation skills (i.e., strategies through which individuals control their expression and experience of positive or negative emotions, [7]). In contrast, *in*validation, which communicates that another is wrong in their description or analyses of their experiences [2], is purported to escalate negative emotional intensity and compromise the therapeutic relationship, therapeutic progress, and client motivation [2, 8–10], and inhibits one’s ability to learn emotion regulation skills [3, 11, 12]. Shenk and Fruzzetti [12] further propose that validation works as a form of emotion regulation itself by minimizing the frequency, intensity, and duration of an emotional response, and ultimately promotes further expression and disclosure of emotion.

Empirical studies support the role of validation in reducing negative emotion. In a study examining the effect of validating and invalidating responses following verbal disclosure of pain, results indicated that participants who received validating responses self-reported significantly less worry and significantly more positive affect following the receipt of these responses than those who received invalidating responses [13]. Similarly, a study examining the effect of validating and invalidating responses in physician interviews following verbal disclosure of back pain found that participants who received validating responses self-reported a significant decrease on all measures of negative affect, pain, and frustration, and higher satisfaction with the physician interview. They also found that those who received validating responses experienced a decline in frustration and anger, compared to an increase in frustration and anger among those who received invalidating responses [14].

Other studies have further explicated the role of (in)validation on emotional response by incorporating physiological indices of emotion. Shenk and Fruzzetti [12] conducted a study examining the effects of validating and invalidating feedback during a math stressor paradigm using self-reported negativity, heart rate, and skin conductance level (SCL) in an undergraduate sample. The authors reported that those who received invalidating feedback experienced a significant increase in negative affect, heart rate, and SCL over the course of the experimental paradigm. Participants who received validating feedback reported nonsignificant changes in negative affect, a significant decline in heart rate, and a significant increase in SCL over the course of the experiment, although the increase in SCL was significantly less steep in comparison to those in the invalidating condition. A second study examined the effects of validating, invalidating, and neutral feedback on self-reported and psychophysiological measurements of emotion following a series of stressor tasks [15]. The authors did not find a significant difference between participants who were validated and those who were given a neutral response; however, participants in the invalidation condition experienced significantly higher levels of heart rate following invalidation compared to individuals in the validation and neutral conditions. These studies collectively suggest that invalidation escalates emotion, whereas validation may decrease emotion or not impact emotion at all.

### (In)validation and emotion specificity

While extant studies have examined the effects of validating and invalidating general negative emotion, what remains unknown is whether the effects of validation or invalidation are differentially impacted depending on the specific emotion that the individual is experiencing. Indeed, understanding the impact of validating or invalidating specific emotions will foster a more nuanced understanding of their emotion sequelae and offer greater insight into how these techniques can be mores strategically used. Although specific emotions may share common features, emotion theorists (e.g., [16]) propose that they are also distinctive and differ on characteristics such as facial expression, physiology, behavioral response, appraisal, regulation strategy, and antecedents. Research further confirms the differences in physiological experience (i.e., [17]), behavioral response (i.e., [18]), antecedents (i.e., [19]), and appraisals (i.e., [20]) for specific negative emotions.

The relationship between validation/invalidation and emotional response may similarly depend on the specific emotion being validated or invalidated. Indeed, the appraisal theory of emotion [21] proposes that some emotions (e.g., happiness, anger, disgust) are associated with certainty or confidence in the perception of one’s thoughts about a situation, and others (e.g., surprise, sadness, fear, shame) are associated with a lack of confidence or certainty about a situation and/or what will happen next [22–24]. As well, research also indicates that emotions tend to differ on their associated action tendency or motivation to avoid or approach stimuli [25]. Certainly, anger is well-studied to be associated with the motivation to approach a stimulus for the purpose of social dominance (i.e., [26, 27]) or aggression (i.e., [28, 29]) whereas fear (i.e., [30–32]), sadness (i.e., [31, 33, 34]), and shame (i.e., [31, 35]) are associated with the motivation to move away or withdraw from the stimulus causing the emotion.

Given the existing literature highlighting conceptual distinctions between specific emotions, it’s possible that validation of emotions linked with greater thought uncertainty and avoidance behaviors (e.g., sadness, shame, fear) lead to enhanced confidence and/or reduced avoidance, thereby decreasing negative emotion. Invalidation of these emotions may potentiate lack of confidence and avoidance and increase negative emotion. In contrast, it is possible that validation of emotions associated with greater thought certainty and approach behaviors (e.g., anger) enhance these processes and increase negative emotion whereas invalidation will have little effect or decrease emotional response. However, as no studies have examined the impact of emotional (in)validation on specific emotions, this theorizing has yet to be tested.

### Emotion dysregulation as a moderator of the impact of (in)validation on emotional response

Another gap in the extant literature is the failure to examine individual differences that may influence the effects of (in)validation on emotional response. Emotion dysregulation (i.e., abnormalities in emotional responding and difficulties with adaptive emotion regulation, [7]) is a transdiagnostic phenomenon implicated in a variety of psychiatric disorders and features, such as mood and anxiety disorders [36]; substance use disorders [37, 38]; suicidality [38]; borderline personality disorder [2]; posttraumatic stress disorder [37]; and eating disorders [39]. Linehan’s [2] Biosocial Model suggests that individuals with a predisposition toward higher emotion dysregulation are a poorer fit for an invalidating caregiving environment, and, with transactions over time, leads to subsequent development of BPD. Thus, according to Linehan [2], individuals with high emotion dysregulation are likely more reactive to invalidation, and especially necessitating (and therefore potentially more responsive to) validation. Individuals with high emotion dysregulation may therefore be particularly likely to exhibit escalations in emotional responses following invalidation, and decreases in it following validation, although it remains unclear if these patterns will be invariant across emotions.

### The present study

Although emerging evidence suggests that validation and invalidation is associated with decreased and increased emotional response, respectively, it is unclear whether there are differential effects on emotional response based on which *specific* emotion is validated or invalidated. Further, it is unknown whether these effects are moderated by emotion dysregulation. Rooted in emotion theories conceptualizing distinctions between core emotions, we hypothesize that validation of fear, sadness, or shame will result in *decreases in negative emotion*, whereas validation of anger will result in *increases in negative emotion*. Given existing theories on the relationship between emotion dysregulation and validation, we hypothesized that emotion dysregulation will potentiate these effects such that higher emotion dysregulation will be associated with even *greater decreases* in negative emotion when fear, sadness, or shame are validated even *greater increases* in negative emotion when anger is validated. Conversely, we hypothesize that, invalidation of fear, sadness, or shame will lead to *increases in negative emotion* and that higher emotion dysregulation would potentiate this effect. The effects of invalidation on anger were exploratory.

## Methods

### Participants

One hundred twenty-six undergraduate students were recruited from an undergraduate psychology research pool at a university in Toronto, ON. Participants provided informed consent prior to their participation in the study and received two credits in their introductory psychology course grade for participating. The study was approved by the university’s Research Ethics Board and was performed in accordance with ethical standards. Participants’ average age was 23.31 (*SD* = 7.84) and 74.6% of them identified as female. The study sample was ethnically diverse, with participants identifying as Black/Black Canadian/Caribbean Origin (15.1%), Asian/Asian Canadian/Pacific Islander (31%), Bi-/Multi-racial (6.3%), Middle Eastern (7.9%), European Origin/White (27.8%), and other (11.9%). Moreover, 72.2% of the sample described their marital status as single/never married, with 17.5%, 7.1%, 2.4%, and 0.8% reporting being in a relationship, married, divorced, and separated, respectively.

### Measures

#### Emotion dysregulation

Emotion dysregulation was measured via the Difficulties with Emotion Regulation Scale (DERS, 40). The DERS is a 39-item scale which measures general emotion dysregulation across six subscales: limited access to emotion regulation strategies, lack of emotional clarity, lack of emotional awareness, difficulties engaging in goal-directed behavior, impulsivity, and nonacceptance of emotional responses. Participants rate the extent to which a series of statements (e.g., “when I’m upset, I feel out of control”) apply to them on a five-point scale ranging from 1 (almost never) to 5 (almost always). Scores are summed and higher scores indicate higher emotion dysregulation. The DERS has strong psychometric properties. For example, the DERS has strong predictive validity, correlating with other behaviors associated with emotion dysregulation, such as self-harm and intimate partner abuse [40]. The DERS also has strong internal reliability, with a Cronbach alpha of 0.94 in the present study.

#### Peak emotion

In order to identify participants’ peak emotion, participants were asked to rate the intensity of 12 emotions immediately following the emotion induction using visual analogue scales ranging from 0 (not at all) to 100 (very): Afraid, anxious, tense, angry, ashamed, rejected, disgusted, guilty, sad, lonely, hopeless, and empty. The emotion that participants rated the highest following the emotion induction was selected as their “peak” emotion and subsequently subsumed under four hierarchical emotion categories for study analyses. Categories of fear (afraid, anxious, tense), sadness (empty, lonely, sad, hopeless), and anger (angry, disgusted) were informed by Shaver, et al.’s [41] hierarchical cluster analysis of emotion words. A combined shame/guilt category (ashamed, rejected, guilt) was constructed given some challenges in the literature in regards to conceptual distinctions between these terms (e.g., [42, 43]) and indications that these terms are not often verbally differentiated and/or are often used interchangeably in colloquial language [35]. Participants who rated the intensity of all emotions as 0 (*n* = 4; 3.2% and *n* = 3; 2.4% for the Validation and Invalidation conditions, respectively) were excluded from the analyses. Table 1 displays the frequency of each peak emotion and their larger groupings for each condition.

**Table 1:** Frequencies of peak emotions reported across conditions

	Frequency: Validation	Frequency: Invalidation
**Fear group**	**36 (28.6%)**	**25 (19.8%)**
Afraid	7 (5.6%)	2 (1.6%)
Anxious	8 (6.3%)	9 (7.1%)
Tense	21 (16.7%)	14 (11.1%)
**Shame group**	**44 (34.9%)**	**44 (34.9%)**
Ashamed	18 (14.3%)	16 (12.7%)
Rejected	18 (14.3%)	20 (15.9%)
Guilty	8 (6.3%)	8 (6.3%)
**Sadness group**	**34 (27.0%)**	**38 (30.2%)**
Empty	7 (5.6%)	12 (9.5%)
Lonely	4 (3.2%)	2 (1.6%)
Sad	14 (11.1%)	11 (8.7%)
Hopeless	9 (7.1%)	13 (10.3%)
**Anger group**	**8 (6.3%)**	**16 (12.7%)**
Angry	5 (4.0%)	13 (10.3%)
Disgusted	3 (2.4%)	3 (2.4%)
**Not included**	**4 (3.2%)**	**3 (2.4%)**

#### Emotion indices: self-report

Self-reported emotion was measured continuously via a rating dial that ranged from 0 (very negative) to 9 (very positive). On the rating dial, participants viewed anchors of “Very negative” under 0, “Very positive” under 9, and “Neutral” under 4. Participants were asked to keep their dominant hand on the rating dial throughout the experiment and to continually move it to reflect changes in their emotional state. Rating dial responses were exported into 10-s segments across the baseline, emotion inductions, and manipulations (i.e., validation or invalidation).

#### Emotion indices: heart rate

Heart rate (HR) and skin conductance level (SCL) were collected using the BIOPAC 6-channel acquisition system (BIOPAC Systems Inc., Model MP150, Goleta, CA). A two-electrode configuration was used with a bioimpedance ground reference module to collect HR data, which was indexed as intervals between R-spikes. Mindware Technologies HRV 2.33 software [44] was used to process HR data, which allowed R-R intervals to be calculated. The identification of R-spikes in Mindware were visually inspected and cleaned for movement artifact and double-scored by study personnel. Participants’ mean HR was exported and analyzed across 30-s segments across the baseline and emotion inductions, and as one 10-s segment during the manipulation (i.e., validation or invalidation).

#### Emotion indices: skin conductance level

Skin conductance level (SCL) was measured via two electrodes placed on the index and middle fingers (medial phalanges) of participant’s nondominant hand [45]. Low- (35 Hz) and high- (0.05 Hz) pass filters were applied and SCL data was digitized at 1,000 samples per second. Participants’ mean SCL were exported into 30-s segments across the baseline, 10-s segments across the emotion inductions, and one 10-s segment during the manipulation (i.e., validation or invalidation).

#### Emotion induction

The emotion induction stimuli were two rejection-themed imagery auditory scripts that involved either (a) the listener’s mother rejecting them because of low exam marks and expressing disappointment or (b) making grave social mistakes during a job interview. The induction lengths were 130 s and 140 s in length, respectively. These stories were played over headphones in second-person narration. Participants are instructed to imagine each script unfolding as if they were really happening to them. Scripts were standardized to contain identical numbers of emotion words, physiological sensations, and thoughts. These scripts were previously piloted in a sample of *N* = 55 undergraduate participants and demonstrated to be emotionally evocative in eliciting distress (Mother script: *F* (1, 54) = 68.18 *p* < 0.001; Job script: *F* (1, 54) = 32.19 *p* < 0.001) and not significantly different with respect to the levels of distress elicited, *t* (54) = 1.83, *p* = 0.07.

### Experimental procedure

Participants were randomly assigned to engage in either the validation or invalidation condition first and script pairing with the validation/invalidation condition was counterbalanced across participants. Thus, each participant listened to both scripts and received both validating and invalidating feedback. Participants were first connected to psychophysiological recording equipment, instructed in the use of the rating dial, and asked to rest quietly without any computer stimuli being presented in a testing room for a 10-min baseline.

Following the baseline, participants then listened to one of the two emotion induction scripts, and were then asked to complete the visual analogue scales used to identify peak emotion. The validation/invalidation manipulation was generated by computer software which extracted the emotion the participant reported as the highest on the visual analogue scales (i.e., “peak emotion”). A sham screen stating “Analyzing Entries” was presented for 10 s during this period. Once the peak emotion was extracted, participants were presented with either validating or invalidating feedback about their peak emotion, depending on their condition. In accordance with Linehan’s definition of validation as communicating that one’s responses “make sense and are understandable” [2], for the validation manipulation, the feedback stated “You reported [PEAK EMOTION] as your most intense emotion. 90% of others reported similarly.” In contrast, for the invalidation manipulation screen, the feedback stated “You reported [PEAK EMOTION] as you’re most intense emotion. Only 10% of others reported similarly.” The manipulation was presented for 10 s. Participants then repeated the induction and manipulation (validation/invalidation) procedure for the other condition. Self-reported and physiological data were collected throughout.

### Data analytic strategy

Hierarchical Linear Modeling (HLM, [46]) was applied using SPSS version 26 software. HLM was selected because this methodology allows for the examination of multilevel data with repeated observations, and fits individual regression slopes, or slopes based on population averages (if only one data point from a participant is collected) to accommodate missing data, allowing maximal statistical power. Random intercept and random slope models were run using restricted maximum likelihood estimation and all person-level variables were entered as fixed effects. Model fits were compared between random intercept, random intercept and random slope (not correlated), and random intercept and random slope (correlated) models, with the model with the lowest Schwarz’s Bayesian Criterion being selected.

Analyses were run separately for the validation and invalidation conditions for each emotion index (i.e., rating dial, HR, and SCL), for a total of six primary analyses. Peak emotion group (dummy coded as follows: Fear = 1, Shame = 2, Sad = 3, Angry = 4) was entered as a between-subjects predictor. Phase (0 = emotion induction; 1 = manipulation) was entered as a within-subjects predictor and the mean-centered total DERS score was entered as a time-invariant predictor. A three-way Peak emotion × Phase × DERS interaction was entered in order to examine whether emotion dysregulation moderated the effect of peak emotion on emotional responses to invalidation or validation. Subsidiary two-way interactions required to build this higher-level, three-way interaction, were also entered into the model. Based on considerations espoused by several statisticians and researchers, we did not apply Bonferroni corrections [47–49]. Namely, we believe that each of our analyses represents a separate test of the null hypothesis. Further, given concerns of compromising the power of the study, we have elected to present all p-values of our primary tests (see Tables 3 and 4) as well as our estimates of fixed effects (see Supplemental Tables) in line with current best practices. In addition, all original data are available upon request to encourage replication and re-analysis by other researchers.

## Results

### Manipulation check

#### Baseline to induction

In order to ensure a stable estimate, only the last 5-min of the baseline was included in analyses of our manipulation check. There were statistically significant main effects of Phase (baseline to induction) for the validation condition across all three outcome measures: rating dial, *F* (1, 3932.41) = 152.11, *p* < 0.01, *β* = -0.56 (*SE* = 0.05), *t* (3932.41) = -12.33, *p* < 0.001; HR, *F* (1, 542.56) = 18.31, *p* < 0.01, *β* = -1.09 (*SE* = 0.26), *t* (542.56) = -4.28, *p* < 0.001; and SCL, *F* (1, 2124.11) = 444.82, *p* < 0.01, *β* = 2.94 (*SE* = 0.14), *t* (2124.11) = 21.09, *p* < 0.001. Similarly, there were statistically significant main effects of Phase (baseline to induction) for the invalidation condition across all three outcome measures: rating dial *F* (1, 3417.32) = 116.28, *p* < 0.01, β = -0.48 (*SE* = 0.05), *t*(3417.32) = -10.78, *p* < 0.001; HR, *F* (1, 609.20) = 25.59, *p* < 0.01, *β* = -1.29 (*SE* = 0.26), *t* (609.20) = -5.06, *p* < 0.001; and SCL, *F* (1, 1180.79) = 259.08, *p* < 0.01, *β* = 2.84 (*SE* = 0.18), *t* (1180.79) = 16.10, *p* < 0.001. These results indicate that, across both conditions, participants demonstrated self-reported increases in negative emotion and physiological changes (i.e., increase in SCL, decrease in HR) from the baseline to the emotion induction, suggesting that the emotion inductions effectively elicited emotional reactivity.

#### Induction to manipulation

There was a statistically significant main effect of Phase (induction to manipulation) for the validation condition for HR, *F* (1, 438.52) = 6.23, *p* = 0.01, *β* = -0.93 (*SE* = 0.37), *t* (438.52) = -2.50, *p* = 0.01, and SCL, *F* (1, 1509.89) = 24.32, *p* < 0.01, *β* = 0.58 (*SE* = 0.12), *t* (1509.89) = 4.93, *p* < 0.001. There was no statistically significant main effect of phase for rating dial, *F* (1, 3501.01) = 2.23, *p* = 0.14, *β* = 0.14 (*SE* = 0.09), *t* (3501.00) = 1.49, *p* = 0.14. There was a statistically significant main effect of Phase (induction to manipulation) for the invalidation condition for rating dial, *F* (1, 1647.18) = 132.63, *p* < 0.01, *β* = 1.12 (*SE* = 0.10), *t*(1647.18) = 11.52, *p* < 0.001 and HR, *F* (1, 428.54) = 4.64, *p* = 0.03, *β* = -0.67 (*SE* = 0.31), *t* (428.54) = -2.15, *p* = 0.03. There was no statistically significant main effect of phase for SCL, *F* (1, 1363.08) = 0.05, *p* = 0.83, SCL, *β* = -0.05 (*SE* = 0.23), *t* (1363.08) = -0.22, *p* = 0.83. These results indicate that participants exhibited physiological changes (i.e., increase in SCL, decrease in HR) when they were validated and exhibited an increase in self-reported positive emotions and a decrease in HR when they were invalidated. See Table 2 for the expected means for each phase (Induction, Manipulation) for the Validation and Invalidation conditions across all indices.

**Table 2:** Expected means for rating dial, heart rate, and skin conductance level for the baseline and each phase within the validation and invalidation conditions

	**Baseline**	**Validation**		**Invalidation**	
		Induction	Manipulation	Induction	Manipulation
**Rating Dial**	4.74	4.07	4.21	4.34	5.46
**Heart Rate**	75.45	73.40	72.47	74.16	73.49
**Skin Conductance Level**	8.71	10.79	11.38	10.14	10.09

Induction = Rejection-based Imagery; Manipulation = Validation or Invalidation; Rating dial range is from 0–9 where 0 = Very Negative and 9 = Very Positive

### Validation condition

#### Rating dial

**Fig. 1:**
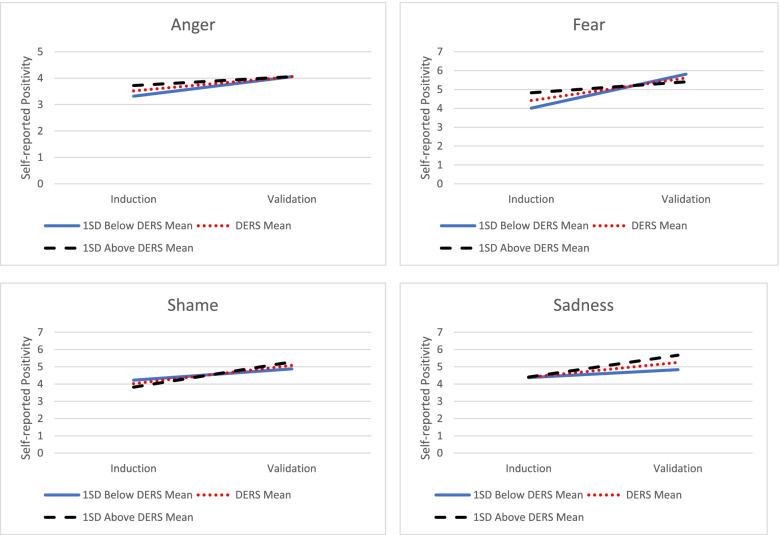
Illustration of Peak Emotion x Phase x Emotion Dysregulation Interaction for Self-reported Positivity in the Validation Condition. DERS = Difficulties with Emotion Regulation Scale. SD = Standard Deviation. Higher emotion dysregulation is associated with greater increases in self-reported positivity for Shame and Sadness and lesser increases in self-reported positivity for Fear. There was not a significant relationship between emotion dysregulation and self-reported positivity for Anger

There was a statistically significant Peak emotion group × Emotion dysregulation × Phase interaction, *F* (3, 1580.17) = 5.26, *p* = 0.001. Higher emotion dysregulation was associated with *greater* increases in positive emotion from the induction to validation when peak emotions were shame, *β* = 0.02, *SE* = 0.01, *t* (1580.83) = 2.26, *p* = 0.02, or sadness, *β* = 0.02, *SE* = 0.007, *t* (1585.52) = 2.59, *p* = 0.01. In contrast, higher emotion dysregulation was associated with *lesser* increases in positive emotion from the induction to validation when peak emotion was fear, *β* = -0.03, *SE* = 0.01, *t* (1583.57) = -2.45, *p* = 0.01. Emotion dysregulation was not associated with changes in emotion when the peak emotion was anger, *β* = -0.01, *SE* = 0.02, *t* (1573.38) = -0.60, *p* = 0.55. See Table 3 and Fig. 1.

**Table 3:** Type III tests of fixed effects for validation condition

	Numerator df	Denominator df	F	*p*-value
Rating Dial				
**Intercept**	**1**	**105.18**	**291.11**	** < .001**
Peak Emotion	3	105.53	.59	.62
**Phase**	**1**	**1584.62**	**51.31**	** < .001**
ED	1	105.67	.47	.49
Peak Emotion x ED	3	105.87	.53	.66
Phase x ED	1	1578.52	.06	.81
Peak Emotion x Phase	3	1584.33	1.01	.39
**Peak Emotion x Phase x ED**	**3**	**1580.17**	**5.26**	**.001**
Heart Rate				
**Intercept**	**1**	**110.03**	**3997.48**	** < .001**
Peak Emotion	3	108.24	.87	.46
**Phase**	**1**	**402.89**	**7.02**	**.008**
**ED**	**1**	**110.86**	**6.41**	**.02**
Peak Emotion x ED	3	106.10	.82	.49
Phase x ED	1	399.19	.07	.80
**Peak Emotion x Phase**	**3**	**403.16**	**2.76**	**.04**
Peak Emotion x Phase x ED	3	400.42	.86	.46
Skin Conductance Level				
**Intercept**	**1**	**102.92**	**146.15**	** < .001**
Peak Emotion	3	102.67	.63	.60
**Phase**	**1**	**1441.20**	**13.19**	** < .001**
ED	1	103.02	.71	.41
Peak Emotion x ED	3	102.64	.16	.92
Phase x ED	1	1441.01	1.98	.16
Peak Emotion x Phase	3	1439.60	2.02	.11
Peak Emotion x Phase x ED	3	1439.84	1.44	.23

Anger is set as Peak Emotion reference group

Phase = 0 is Induction Period, Phase = 1 is Validation Period

*p* < .05 effects are bolded

ED = Mean-centered total scores on Difficulties with Emotion Regulation Scale (DERS)

Significant effects are bolded

#### Heart rate

**Fig. 2:**
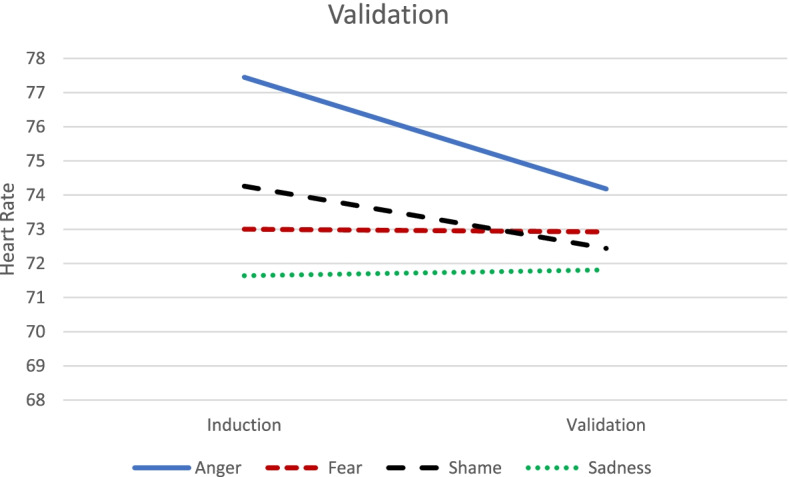
Illustration of Peak Emotion x Phase Interaction for Heart Rate in the Validation Condition. There were significant reductions in heart rate from the induction to validation when the peak emotion was Anger and Shame, but no significant changes in heart rate when the peak emotion was Fear or Sadness

There was no statistically significant Peak emotion group x Emotion dysregulation x Phase interaction *F* (3, 400.42) = 0.86, *p* = 0.46. However, there was a statistically significant Peak emotion group × Phase interaction, *F* (3, 403.16) = 2.76, *p* = 0.04. There were reductions in HR from the induction to validation when the peak emotion was anger, β = -3.27, SE = 1.46, *t* (402.54) = -2.24, *p* = 0.03, and shame, *β* = -1.82, SE = 0.64, *t* (404.48) = -2.86, *p* = 0.004, and no statistically significant changes in HR when the peak emotion was fear, *t* (402.79) = -0.11, *p* = 0.91, or sadness, *t* (403.17) = 0.24, *p* = 0.81. See Table 3 and Fig. 2.

#### Skin conductance level

There was no statistically significant Peak emotion group Emotion dysregulation × Phase interaction, *F* (3,1439.84) = 1.44, *p* = 0.23. Subsidiary 2-way interactions were also non-significant. See Table 3.

### Invalidation condition

#### Rating dial

**Fig. 3:**
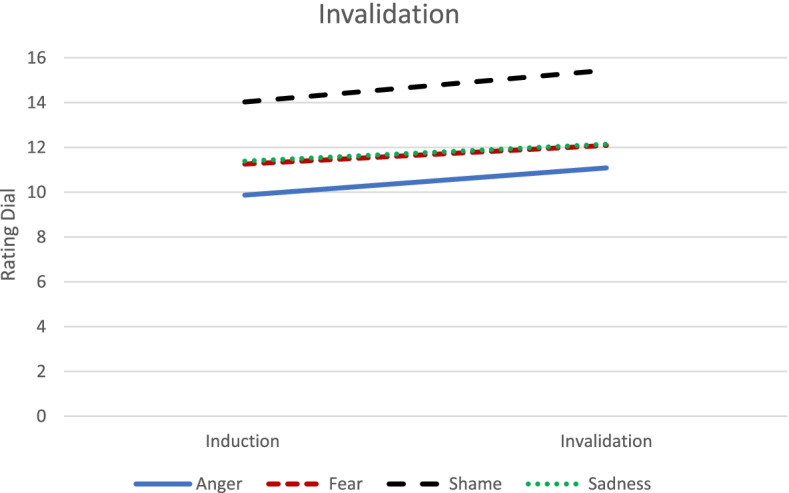
Illustration of Peak Emotion x Phase Interaction for Rating Dial in the Invalidation Condition. There were increases in positive emotion from the induction to invalidation for all emotions; however, there were significantly greater increases in positive emotion in shame versus fear, and shame versus sadness

There was no statistically significant Peak emotion group × Emotion dysregulation × Phase interaction on rating dial, *F* (3, 1544.02) = 1.78, *p* = 0.15. However, there was a statistically significant Peak emotion group x Phase interaction, *F* (3, 1544.36) = 3.02, *p* = 0.03. Examination of fixed effects indicated that, while there were increases in positive emotion from the induction to invalidation for all emotions (anger, *β* = 1.22, *SE* = 0.29, *t* (1546.35) = 4.19, *p* < 0.001; fear, *β* = 0.83, *SE* = 0.23, *t* (1543.15) = 3.60, *p* < 0.001; shame, *β* = 1.42, *SE* = 0.16, *t* (1542.87) = 8.80, *p* < 0.001; sadness *β* = 0.76, *SE* = 0.18, *t* (1544.56) = 4.21, *p* < 0.001) there were statistically significantly greater increases in positive emotion in shame versus fear (*p* = 0.04), and shame versus sadness (*p* = 0.006). See Table 4 and Fig. 3.

**Table 4:** Type III tests of fixed effects for invalidation condition

	Numerator df	Denominator df	F	*p*-value
Rating Dial				
**Intercept**	**1**	**112.58**	**293.44**	** < .001**
Peak Emotion	3	111.25	2.05	.11
**Phase**	**1**	**1544.72**	**91.11**	** < .001**
ED	1	113.21	.16	.69
Peak Emotion x ED	3	110.16	.56	.64
Phase x ED	1	1544.82	1.06	.30
**Peak Emotion x Phase**	**3**	**1544.36**	**3.02**	**.03**
Peak Emotion x Phase x ED	3	1544.02	1.775	.15
Heart Rate				
**Intercept**	**1**	**102.91**	**4510.11**	** < .001**
Peak Emotion	3	102.95	1.42	.24
**Phase**	**1**	**403.82**	**4.38**	**.04**
ED	1	102.89	2.01	.16
Peak Emotion x ED	3	102.61	.26	.86
Phase x ED	1	402.96	.15	.70
Peak Emotion x Phase	3	404.02	.79	.50
Peak Emotion x Phase x ED	3	403.39	.14	.94
Skin Conductance Level				
**Intercept**	**1**	**98.91**	**128.01**	** < .001**
Peak Emotion	3	99.42	.41	.75
Phase	1	1294.61	.02	.88
ED	1	99.24	.41	.52
Peak Emotion x ED	3	100.08	.03	.99
Phase x ED	1	1284.81	.29	.59
Peak Emotion x Phase	3	1298.60	1.57	.19
Peak Emotion x Phase x ED	3	1293.70	.33	.80

Anger is set as Peak Emotion reference group

Phase = 0 is Induction Period, Phase = 1 is Invalidation Period

*p* < .05 effects are bolded

ED = Mean-centered total scores on Difficulties with Emotion Regulation Scale (DERS)

Significant effects are bolded

#### Heart rate

There was no statistically significant Peak emotion group x Emotion dysregulation x Phase interaction on HR, *F* (3, 403.39) = 0.14, *p* = 0.94. However, there was a statistically significant effect of Phase, *F* (3, 403.82) = 4.38, *p* = 0.04 such that there was a reduction in HR from the Induction to Invalidation condition when peak emotion was Anger and at mean emotion dysregulation levels. See Table 4.

#### Skin conductance level

There was no statistically significant Peak emotion group x Emotion dysregulation x Phase interaction on SCL, *F* (3, 1293.70) = 0.33, *p* = 0.80. Subsidiary 2-way interactions were also non-significant. See Table 4.

## Discussion

While emerging evidence and substantial theory point to validation as a precursor to decreased emotional intensity and invalidation as a precursor to increased emotional intensity, it is yet unclear whether these relationships are invariant across negative emotion categories. In line with theoretical underpinnings positing conceptual distinctions between different emotions, the present study hypothesized that fear, sadness, and shame would reflect previous research demonstrating reductions in negative emotional intensity when validated and increases in negative emotional intensity when invalidated. We further hypothesized that emotion dysregulation would potentiate these effects. Conversely, we hypothesized that anger would be associated with increases in negative emotional intensity in response to validation.

### Validation

Results of the validation condition were in partial support of our hypotheses. Higher levels of emotion dysregulation were associated with greater increases in self-reported positive emotion if the peak emotion reported was shame or sadness. However, the opposite effect emerged for fear, such that higher emotion dysregulation was associated with *lesser* increases in self-reported positive emotion. Perhaps emotions associated with rumination and a retrospective focus – such as sadness and shame– [50, 51] are more sensitive to validation than emotions associated with future-related uncertainty, such as fear or anxiety. In addition, it is possible that shame, which is a painful sense that the self is wrong or inferior [52], is most responsive to an outside observer providing an individual with a sense of similarity and commiseration with the general population, thus reducing feelings of isolation in perceived self-defect. This explanation can logically be extended to the sadness group, which exhibited a similar pattern to shame. In the case of sadness, thoughts associated with past failures, fundamental defects in the self, and loss may have been triggered by the rejection-related emotion induction. Once again, that validation communicates a common experience of such feelings may have had a specifically salient effect on those in the sadness group, and those who have particularly intense emotions (i.e., high emotion dysregulation).

Conversely, increases in positive emotional intensity following validation was *attenuated* among those with higher levels of emotion dysregulation. It is unclear whether these findings relate to the experience of fear itself, or an individual difference related to those most likely to rate fear as their peak emotional experience during this type of task. It may be that the future anticipation of a negative event or consequence is less likely to be assuaged by knowing that others similarly anticipate the negative event. Indeed, it could be that validation of fear, rather than providing a comforting notion of a common experience as in shame or sadness, is less likely to mitigate distress by seeming to confirm the presence of a threat. In this case, potential rises in distress from the perceived confirmation of a threatening stimuli may obstruct otherwise comforting effects of validation.

Importantly, in contrast to the moderating effects of emotion dysregulation on self-reported intensity, the lack of significant 3-way interactions in the physiological outcomes indicate that emotion dysregulation does not appear to moderate physiological effects of validation. However, there were differential effects of validation on HR depending on the peak emotion categories. Namely, at average levels of emotion dysregulation, validation of anger and shame (but not fear or sadness) was associated with reductions in HR; in contrast, there were no corresponding changes in SCL for any of the emotions. The discordance between the HR and SCL findings might indicate that the reductions in HR were perhaps mediated by parasympathetic activity rather than sympathetic activity. As well, given that reductions in HR have been linked with an orienting response [53], it’s possible that HR reductions in the anger and shame groups indicate increased attention while receiving the validating feedback.

### Invalidation

In contrast to our findings from the validation condition, we did not find a moderating effect of emotion dysregulation on any peak emotion for any of our indices. Interestingly, our manipulation check examining changes from the induction to the invalidation period indicated that participants exhibited a significant decrease in HR and an *increase* in positive emotions. Consistently, our Peak emotion group x Phase interaction on rating dial indicated an increase in positivity across all four peak emotions examined, and significantly greater increases in positivity for those reporting peak shame compared to both fear and sadness. Perhaps receiving invalidating feedback was, in fact, slightly alleviating relative to the imagery induction, and sparked interest or surprise in participants.

In addition, it is important to note that the interval during which participants were invalidated was rather brief (i.e., 10 s). This is in contrast to previous validation/invalidation experimental studies [12, 15] in which investigators typically examined longer periods (e.g., 30 min.) during which participants received repeated rounds of validating or invalidating feedback interspersed throughout the completion of challenging tasks. However, given that these studies examined change in emotions over the course of the entire task rather than specific to the periods in which validating or invalidating feedback was presented, the effects of “pure” validation/invalidation from these studies are unclear. Such methodological discrepancies may account for why these studies found escalations in physiological response (e.g., HR) in response to invalidation but our study did not. However, it is worth noting that we did find effects in the validation condition, indicating that the emotion sequelae of validation versus invalidation may emerge on different trajectories.

### Limitations and future directions

Our study is limited by the examination of a convenience sample of undergraduates. Second, although the number of participants binned into the Fear, Shame, and Sadness categories were relatively comparable, the number of participants binned into the Anger group was small, therefore creating imbalanced groups in our analyses. In addition, although our study examined self-reported changes in general negative emotion in response to validation/invalidation, we did not examine self-reported changes in the *specific* emotion that was validated or invalidated. Thus, it is also important to note that our focus on the change in emotional intensity may be artificially masking the improvement or worsening of specific emotions. The use of a more ecologically, personally-relevant task might provide different results. Further, because our task was not only standardized across participants, but also imaginal in nature, it is unclear how results would be altered in the presence of an in vivo emotion elicitation.

## Conclusions

Overall, this study provides important insight regarding the effect of validation on specific emotional experiences. Primarily, among individuals with higher levels of emotion dysregulation, validation of emotions related negative views of the self (e.g., shame, sadness), may be particularly potent in reducing general negative emotional intensity or increasing positive emotional experiencing. Indeed, the use of validation might be in contrast to therapeutic techniques that seek to initially challenge the self-deprecating beliefs typically associated with these emotions. Our findings therefore suggest that the implementation of validation as a potential emotion regulation technique before using a more “challenging” strategy such as cognitive restructuring might be particularly effective for those higher in emotion dysregulation. In contrast, for these individuals, validation may be less likely to improve feelings of fear and anxiety. In those cases, direct challenging or exposure may be most beneficial. Although our examination of the moderating effects of emotion dysregulation on validation/invalidation offer some important clinical implications, more research is needed to directly test these hypotheses as they apply to clinical intervention.

## Supplementary Information

**Additional file 1:** **Supplemental Table 1.** Estimates of Fixed Effects for Validation Condition: Rating Dial. **Supplemental Table 2.** Estimates of Fixed Effects for Validation Condition: Heart Rate. **Supplemental Table 3.** Estimates of Fixed Effects for Validation Condition: Skin Conductance Level. **Supplemental Table 4.** Estimates of Fixed Effects for Invalidation Condition: Rating Dial. **Supplemental Table 5.** Estimates of Fixed Effects for Invalidation Condition: Heart Rate. **Supplemental Table 6.** Estimates of Fixed Effects for Invalidation Condition: Skin Conductance Level.

## Data Availability

The datasets used and/or analyzed during the current study are available from the corresponding author on reasonable request.

## Abbreviations

HR: Heart rate
SCL: Skin conductance level
BPD: Borderline personality disorder
DBT: Dialectical behavior therapy
DERS: Difficulties with Emotion Regulation Scale
HLM: Hierarchical linear modeling
