# Supplementary material for: The who and what of validation: an experimental examination of validation and invalidation of specific emotions and the moderating effect of emotion dysregulation
Source: Borderline Personal Disord Emot Dysregul. 2022 May 18;9:15. doi: 10.1186/s40479-022-00185-x (PMC9116024; doi:10.1186/s40479-022-00185-x)
Supplement: Supplementary file 1 — Additional file 1: Supplemental Table 1. Estimates of Fixed Effects for Validation Condition: Rating Dial. Supplemental Table 2. Estimates of Fixed Effects for Validation Condition: Heart Rate. Supplemental Table 3. Estimates of Fixed Effects for Validation Condition: Skin Conductance Level. Supplemental Table 4. Estimates of Fixed Effects for Invalidation Condition: Rating Dial. Supplemental Table 5. Estimates of Fixed Effects for Invalidation Condition: Heart Rate. Supplemental Table 6. Estimates of Fixed Effects for Invalidation Condition: Skin Conductance Level. [file 40479_2022_185_MOESM1_ESM.docx]

Supplemental Table 1.

*Estimates of Fixed Effects for Validation Condition: Rating Dial*

|  | β | *SE* | df | t | *p-value* |
| --- | --- | --- | --- | --- | --- |
| **Intercept** | **3.52** | **.75** | **104.46** | **4.71** | **<.001** |
| Fear | .90 | .83 | 105.15 | 1.08 | .28 |
| Shame | .50 | .81 | 104.55 | .62 | .54 |
| Sadness | .86 | .83 | 104.68 | 1.04 | .30 |
| Anger | - | - | - | - | - |
| Phase | .54 | .40 | 1584.98 | 1.36 | .17 |
| ED | .01 | .03 | 102.74 | .50 | .62 |
| Fear × ED | .009 | .04 | 104.85 | .25 | .80 |
| Shame x ED | -.02 | .04 | 104.26 | -.68 | .50 |
| Sadness x ED | -.01 | .03 | 104.03 | -.43 | .67 |
| Anger x ED | - | - | - | - | - |
| Phase × ED | -.01 | .02 | 1573.38 | -.60 | .55 |
| Fear × phase | .65 | .44 | 1584.94 | 1.47 | .14 |
| Shame x phase | .51 | .43 | 1584.76 | 1.19 | .24 |
| Sadness x phase | .31 | .44 | 1584.76 | .71 | .48 |
| Anger x phase | - | - | - | - | - |
| Fear × ED × phase | -.02 | .02 | 1576.78 | -.89 | .37 |
| Shame x ED x phase | .03 | .02 | 1575.69 | 1.73 | .08 |
| Sadness x ED x phase | .03 | .02 | 1575.57 | 1.63 | .10 |
| Anger x ED x phase | - | - | - | - | - |

*Note.* Anger is set as Peak Emotion reference group. Phase = 0 is Induction Period, Phase = 1 is Validation Period.  *p* < .05 effects are bolded. ED = Mean-centered total scores on Difficulties with Emotion Regulation Scale (DERS). Significant effects are bolded.

Supplemental Table 2.

*Estimates of Fixed Effects for Validation Condition: Heart Rate*

|  | β | *SE* | df | t | *p-value* |
| --- | --- | --- | --- | --- | --- |
| **Intercept** | **77.45** | **3.66** | **111.07** | **21.14** | **<.001** |
| Fear | -4.44 | 4.07 | 110.31 | -1.09 | .27 |
| Shame | -3.19 | 3.98 | 110.86 | -.80 | .43 |
| Sadness | -5.81 | 4.04 | 110.86 | -1.43 | .16 |
| Anger | - | - | - | - | - |
| **Phase** | **3.27** | **1.46** | **402.54** | **-2.24** | **.03** |
| ED | .29 | .15 | 109.98 | 1.92 | .06 |
| Fear × ED | -.25 | .18 | 111.63 | -1.36 | .18 |
| Shame x ED | -.14 | .18 | 111.58 | -.76 | .45 |
| Sadness x ED | -.23 | .17 | 111.23 | -1.37 | .17 |
| Anger x ED | - | - | - | - | - |
| Phase × ED | .04 | .06 | 394.28 | .69 | .49 |
| Fear × phase | 3.19 | 1.63 | 402.59 | 1.95 | .05 |
| Shame x phase | 1.46 | 1.59 | 402.85 | .91 | .36 |
| **Sadness x phase** | **3.44** | **1.62** | **402.66** | **2.13** | **.04** |
| Anger x phase | - | - | - | - | - |
| Fear × ED × phase | -.04 | .07 | 397.08 | -.50 | .62 |
| Shame x ED x phase | -.09 | .07 | 397.63 | -1.24 | .22 |
| Sadness x ED x phase | -.02 | .07 | 395.64 | -.24 | .81 |
| Anger x ED x phase | - | - | - | - | - |

*Note.* Anger is set as Peak Emotion reference group. Phase = 0 is Induction Period, Phase = 1 is Validation Period.  *p* < .05 effects are bolded. ED = Mean-centered total scores on Difficulties with Emotion Regulation Scale (DERS). Significant effects are bolded.

Supplemental Table 3.

*Estimates of Fixed Effects for Validation Condition: Skin Conductance Level*

|  | β | *SE* | df | t | *p-value* |
| --- | --- | --- | --- | --- | --- |
| **Intercept** | **12.11** | **2.85** | **103.27** | **4.26** | **<.001** |
| Fear | .06 | 3.17 | 103.01 | .02 | .99 |
| Shame | -2.29 | 3.11 | 103.15 | -.74 | .46 |
| Sadness | -1.71 | 3.17 | 103.17 | -.54 | .59 |
| Anger | - | - | - | - | - |
| Phase | .43 | .47 | 1443.21 | .91 | .36 |
| ED | .01 | .12 | 104.25 | .10 | .92 |
| Fear × ED | .07 | .14 | 103.47 | .51 | .61 |
| Shame x ED | -.001 | .14 | 103.56 | -.007 | .99 |
| Sadness x ED | .03 | .13 | 103.86 | .22 | .83 |
| Anger x ED | - | - | - | - | - |
| Phase × ED | -.003 | .02 | 1445.17 | -.15 | .88 |
| Fear × phase | -.27 | .52 | 1442.39 | -.51 | .61 |
| Shame x phase | .45 | .52 | 1442.00 | .89 | .38 |
| Sadness x phase | .31 | .52 | 1442.50 | .59 | .56 |
| Anger x phase | - | - | - | - | - |
| Fear × ED × phase | .03 | .02 | 1443.08 | 1.37 | .17 |
| Shame x ED x phase | .02 | .02 | 1441.49 | .84 | .40 |
| Sadness x ED x phase | .0002 | .02 | 1444.65 | .009 | .99 |
| Anger x ED x phase | - | - | - | - | - |

*Note.* Anger is set as Peak Emotion reference group. Phase = 0 is Induction Period, Phase = 1 is Validation Period.  *p* < .05 effects are bolded. ED = Mean-centered total scores on Difficulties with Emotion Regulation Scale (DERS). Significant effects are bolded.

Supplemental Table 4.

*Estimates of Fixed Effects for Invalidation Condition: Rating Dial*

|  | β | *SE* | df | t | *p-value* |
| --- | --- | --- | --- | --- | --- |
| **Intercept** | **9.87** | **1.80** | **118.21** | **5.48** | **<.001** |
| Fear | 1.39 | 2.28 | 113.89 | .61 | .54 |
| **Shame** | **4.16** | **2.05** | **115.42** | **2.03** | **.05** |
| Sadness | 1.52 | 2.11 | 116.42 | .72 | .47 |
| Anger | - | - | - | - | - |
| Phase | 1.22 | .29 | 1546.35 | 4.19 | <.001 |
| ED | -.06 | .11 | 121.65 | -.54 | .59 |
| Fear × ED | .01 | .13 | 115.38 | .11 | .91 |
| Shame x ED | .11 | .12 | .117.62 | .94 | .35 |
| Sadness x ED | .04 | .11 | 119.70 | .38 | .71 |
| Anger x ED | - | - | - | - | - |
| Phase × ED | -.03 | .02 | 1546.84 | -1.67 | .10 |
| Fear × phase | -.39 | .37 | 1545.11 | -1.04 | .30 |
| Shame x phase | .21 | .33 | 1545.53 | .62 | .54 |
| Sadness x phase | -.46 | .34 | 1545.85 | -1.34 | .18 |
| Anger x phase | - | - | - | - | - |
| Fear × ED × phase | .04 | .02 | 1545.56 | 1.78 | .08 |
| Shame x ED x phase | .02 | .02 | 1545.67 | .88 | .38 |
| Sadness x ED x phase | .03 | .02 | 1546.35 | 1.82 | .07 |
| Anger x ED x phase | - | - | - | - | - |

*Note.* Anger is set as Peak Emotion reference group. Phase = 0 is Induction Period, Phase = 1 is Validation Period.  *p* < .05 effects are bolded. ED = Mean-centered total scores on Difficulties with Emotion Regulation Scale (DERS). Significant effects are bolded.

Supplemental Table 5.

*Estimates of Fixed Effects for Invalidation Condition: Heart Rate*

|  | β | *SE* | df | t | *p-value* |
| --- | --- | --- | --- | --- | --- |
| **Intercept** | **68.74** | **2.81** | **101.36** | **24.45** | **<.001** |
| Fear | 6.54 | 3.66 | 102.57 | 1.79 | .08 |
| Shame | 6.32 | 3.24 | 102.40 | 1.96 | .05 |
| Sadness | 4.94 | 3.31 | 1101.82 | 1.49 | .14 |
| Anger | - | - | - | - | - |
| Phase | -1.30 | .94 | 402.98 | -1.38 | .17 |
| ED | .010 | .16 | 99.30 | .06 | .95 |
| Fear × ED | .18 | .22 | 102.13 | .84 | .41 |
| Shame x ED | .07 | .18 | 101.60 | .36 | .72 |
| Sadness x ED | .06 | .17 | 100.47 | .36 | .72 |
| Anger x ED | - | - | - | - | - |
| Phase × ED | .007 | .05 | 400.90 | .13 | .90 |
| Fear × phase | 1.52 | 1.22 | 403.60 | 1.25 | .21 |
| Shame x phase | .19 | 1.08 | 403.56 | .17 | .86 |
| Sadness x phase | .47 | 1.11 | 403.12 | .42 | .68 |
| Anger x phase | - | - | - | - | - |
| Fear × ED × phase | -.01 | .07 | 402.58 | -.18 | .86 |
| Shame x ED x phase | -.00003 | .06 | 401.81 | .00 | 1.00 |
| Sadness x ED x phase | .02 | .06 | 401.34 | .28 | .78 |
| Anger x ED x phase | - | - | - | - | - |

*Note.* Anger is set as Peak Emotion reference group. Phase = 0 is Induction Period, Phase = 1 is Validation Period.  *p* < .05 effects are bolded. ED = Mean-centered total scores on Difficulties with Emotion Regulation Scale (DERS). Significant effects are bolded.

Supplemental Table 6.

*Estimates of Fixed Effects for Invalidation Condition: Skin Conductance Level*

|  | β | *SE* | df | t | *p-value* |
| --- | --- | --- | --- | --- | --- |
| **Intercept** | **12.26** | **2.36** | **95.67** | **5.19** | **<.001** |
| Fear | -2.63 | 3.05 | 98.17 | -.86 | .39 |
| Shame | -1.53 | 2.75 | 97.17 | -.56 | .58 |
| Sadness | -2.86 | 2.85 | 96.93 | -1.01 | .32 |
| Anger | - | - | - | - | - |
| Phase | .59 | .67 | 1272.33 | .88 | .38 |
| ED | .06 | .14 | 94.24 | .41 | .68 |
| Fear × ED | -.02 | .18 | 98.52 | -.12 | .91 |
| Shame x ED | -.04 | .17 | 96.19 | -.24 | .81 |
| Sadness x ED | -.04 | .15 | 95.50 | -.24 | .81 |
| Anger x ED | - | - | - | - | - |
| Phase × ED | -.002 | .04 | 1254.57 | -.04 | .97 |
| Fear × phase | -1.05 | .86 | 1288.71 | -1.22 | .22 |
| Shame x phase | -1.12 | .78 | 1283.71 | -1.44 | .15 |
| Sadness x phase | -.04 | .81 | 1280.95 | -.05 | .96 |
| Anger x phase | - | - | - | - | - |
| Fear × ED × phase | -.002 | .05 | 1271.61 | -.03 | .97 |
| Shame x ED x phase | -.03 | .05 | 1274.07 | -.57 | .57 |
| Sadness x ED x phase | .003 | .04 | 1264.79 | .07 | .94 |
| Anger x ED x phase | - | - | - | - | - |

*Note.* Anger is set as Peak Emotion reference group. Phase = 0 is Induction Period, Phase = 1 is Validation Period.  *p* < .05 effects are bolded. ED = Mean-centered total scores on Difficulties with Emotion Regulation Scale (DERS). Significant effects are bolded.
